# Effects of emotional study context on immediate and delayed recognition memory: Evidence from event-related potentials

**DOI:** 10.3758/s13415-021-00944-3

**Published:** 2021-09-08

**Authors:** Lisa Katharina Kuhn, Regine Bader, Axel Mecklinger

**Affiliations:** 1 Present Address: National Foundation for Educational Research, Centre for Assessment, Slough, SL1 2DQ UK; 2 Experimental Neuropsychology Unit, Department of Psychology, Saarland University, 66123 Saarbruecken, Germany

**Keywords:** Emotion, Familiarity, Recollection, ERP, Episodic memory

## Abstract

Whilst research has largely focused on the recognition of emotional items, emotion may be a more subtle part of our surroundings and conveyed by context rather than by items. Using ERPs, we investigated which effects an arousing context during encoding may have for item-context binding and subsequent familiarity-based and recollection-based item-memory. It has been suggested that arousal could facilitate item-context bindings and by this enhance the contribution of recollection to subsequent memory judgements. Alternatively, arousal could shift attention onto central features of a scene and by this foster unitisation during encoding. This could boost the contribution of familiarity to remembering. Participants learnt neutral objects paired with ecologically highly valid emotional faces whose names later served as neutral cues during an immediate and delayed test phase. Participants identified objects faster when they had originally been studied together with emotional context faces. Items with both neutral and emotional context elicited an early frontal ERP old/new difference (200-400 ms). Neither the neurophysiological correlate for familiarity nor recollection were specific to emotionality. For the ERP correlate of recollection, we found an interaction between stimulus type and day, suggesting that this measure decreased to a larger extend on Day 2 compared with Day 1. However, we did not find direct evidence for delayed forgetting of items encoded in emotional contexts at Day 2. Emotion at encoding might make retrieval of items with emotional context more readily accessible, but we found no significant evidence that emotional context either facilitated familiarity-based or recollection-based item-memory after a delay of 24 h.

## Introduction

Often, formative memories from our past tend to be very emotional, or we seem to remember emotional memories particularly well. Indeed, previous literature has established a reliable influence of emotional arousal on our ability to recall events. Consistently, emotional items have been found to be remembered better than neutral items (Kensinger, 2009; Mather & Sutherland, 2011; Payne et al., 2008; Yonelinas & Ritchey, 2015). It is possible that this emotion advantage takes place during perceptual processing and encoding highly arousing stimuli may be prioritised for processing (Mather & Sutherland, 2011; Pessoa & Ungerleider, 2004). For example, the inability to detect a second target immediately after the presentation of a first target during an attentional blink paradigm is less prominent when the second target is highly arousing (Anderson, 2005). Neuroimaging data further suggest enhanced activity in the visual cortex (Vuilleumier et al., 2004) and amygdala (Mather et al., 2004) during the presentation of emotional versus neutral stimuli, which implies a rapid and automatic prioritisation of arousing stimuli and could explain why emotional items are subsequently remembered better than neutral items.

However, what becomes evident is that consolidation of emotional stimuli takes time: the actual emotion advantage may only take effect and be measurable after a delay. Some early reports suggest that arousal incrementally supports learning after a short consolidation period ranging from 6 minutes (Kaplan & Kaplan, 1969) to 20-40 minutes after encoding (Kleinsmith et al., 1963). Others reported a clear emotion advantage emerging typically 24 h after encoding (Sharot & Phelps, 2004; Sharot & Yonelinas, 2008), whereas others again report longer-lasting emotional memory enhancements of up to 1 week (Ritchey et al., 2008; Ventura-Bort et al., 2016) or even 1 year (Dolcos et al., 2005; Weymar et al., 2011). Neuroimaging studies have linked this emotion advantage to interactions between the amygdala and medial temporal lobe regions (Dolcos et al., 2012) and the “emotional binding account” (Yonelinas & Ritchey, 2015) proposes that arousing stimuli engage amygdala activity during encoding, which in turn supports more common hippocampal binding mechanisms. The model suggests that these item-emotion bindings are forgotten slower than the item-context bindings, which are processed by the hippocampus alone because of the different properties of neural structures that are involved in the underlying binding mechanisms. For example, a high rate of cell death and neurogenesis in the hippocampus affects how quickly neutral items will be forgotten. Based on animal studies, it is plausible that because emotional items are remembered under amygdala-support, which has a slower rate of cell-death than the hippocampus and therefore a slower forgetting-rate, these emotional items may be forgotten slower than neutral items (Akers et al., 2014). Alternatively, Yonelinas and Ritchey (2015) postulated that “the amygdala may simply be more resistant to interference, because there are typically fewer competing emotional experiences outside of the experimental context, relative to neutral experiences” (p. 7). Long-term memory consolidation of emotional arousing events further commonly involves adrenal stress hormones, such as epinephrine and corticosterone, in interaction with the activation of the basolateral region of the amygdala (Buchanan & Adolphs, 2003; Gold & McGaugh, 1975; McGaugh, 2004, 2013). For example, long-term memory for emotional pictures was improved when cortisol levels were enhanced during encoding (Buchanan & Lovallo, 2001), implying that cortisol mediates the interaction between arousal and long-term storage.

### Influence of emotional binding on item memory

However, an item is rarely remembered in isolation. Whilst emotional stimuli often are better remembered than neutral stimuli when the stimulus is a single item (item-emotion binding, such as for emotional photos, in Sharot & Yonelinas, 2008), the influence of emotional context on item memory is less clear and little research has been conducted investigating this link. Some studies have investigated what effect emotional items have on the memory for their study context, and the results have been mixed at best. For example, some report that emotional items can improve recognition memory for the context (e.g., location memory for arousing pictures, in Mather et al., 2009), whereas others reported that the emotional item takes attention away from peripheral context and therefore impairs context memory (Bennion et al., 2017; Mao et al., 2015). Others report no emotion effect on context memory at all (Sharot & Yonelines, 2008).

However, in real-life, emotional cues may be more subtle. We may encounter situations where the context of an item carries the emotional load rather than the actual item itself. The question arises to what extend the emotional context may influence our memory for that specific item or event. For example, we may remember a seemingly neutral encounter with a dog very differently if this has been accompanied by a bystander’s fearful scream. In this situation, the emotional context (i.e., the scream) may boost future memory for a neutral cue (i.e., the dog). Indeed, recent studies have reported enhanced memory for items that were paired with emotional rather than neutral contexts (Maratos & Rugg, 2001; Ritchey et al., 2013; Smith et al., 2006; Ventura-Bort et al., 2016), suggesting that emotional arousal transmitted onto an item via its emotional context (such as neutral words within a negatively valenced sentence, in Maratos & Rugg, 2001) can indeed boost item memory under certain circumstances.

### Neurophysiological correlates of emotional item memory

Dual process models suggest that there are two distinct subcomponents of recognition memory—familiarity and recollection, which differ based on the perceived strength and quality of individuals’ recognition judgements. This may range from a mere feeling of familiarity (i.e., “familiarity” component) to remembering specific contextual details as for example bindings between specific objects of a scene as reflected by the recollection component (Mecklinger, 2000; Rugg, 1995; Yonelinas, 1995). It has been suggested that emotion improves item memory selectively, with emotion and arousal affecting recollection processes, but not familiarity for individual (mostly negatively loaded) items (Anderson et al., 2006; LaBar & Phelps, 1998; Ochsner, 2000; Sharot & Yonelinas, 2008; Yonelinas & Ritchey, 2015). For example, *remember/know* procedures have indicated a memory advantage for emotional over neutral materials that were *remembered*, but this pattern was not true for familiarity-related *know* responses (Johansson et al., 2004; Kensinger & Corkin, 2003).

Whilst the studies above were based on meta-memory judgements, ERPs provide a means to additionally assess neurophysiological markers of cognition with high temporal resolution. Existing ERP studies on recognition memory revealed that recollection and familiarity have unique ERP signatures. The ERP correlate of recollection is the parietal old/new effect (see Rugg & Curran, 2007, or Friedman & Johnson, 2000, for a review). It takes the form of more positive going waveforms for hits than correct rejections between 500 and 700 ms, which are most pronounced at (left) parietal recording sites. Interestingly, this parietal old/new effect for recollection has previously been found to be modulated by emotion (Maratos & Rugg, 2001; Ventura-Bort et al., 2016; Weymar et al., 2011; Wirkner et al., 2018), for example with a parietal old/new effect for emotional, negatively-valenced, but not neutral faces (Johansson et al., 2004).

The putative ERP correlate of familiarity is the mid-frontal old/new effect (also called FN400 or FN400 old/new effect). It takes the form of more positive going ERP waveforms for hits than correct rejections and is most pronounced between 300 and 500 ms at frontal recording sites (Mecklinger, 2000; for reviews see Mecklinger, 2006; Rugg & Curran, 2007; Mecklinger & Bader 2020; but see Paller et al., 2007; Voss & Federmeier, 2011 for alternative interpretations of the FN400). Consistent with the view that the mid-frontal old/new effect is associated with familiarity memory, the magnitude of this effect co-varies with familiarity strength, operationalised as confidence with which recognition decisions are given (Woodruff et al., 2006; Sarah & Rugg, 2010). The magnitude of the FN400 can further be influenced by manipulating the degrees of familiarity, for example by changing the response bias (Azimian-Faridani & Wilding, 2006) or test-format (Bader et al., 2020). Of note, this early old/new effect has not been modulated by emotionality (Johansson et al., 2004; Windman & Kutas, 2001). It is therefore possible that there is a dissociation in ERP correlates for the recognition of emotional items, with a contribution of emotion to recollection but not to familiarity-based remembering. As such, there would be similar levels of familiarity between emotional and neutral hits—comparable to what was shown in behavioural studies.

### Neurophysiological correlates of binding emotional context to items

For the influence of emotional context on item memory, existing evidence has been mixed and this inevitably introduces ambiguity about the underlying processes that may support memory performance of emotional episodes. Some studies that have previously reported an advantage for items that were bound to emotional context have linked the underlying mechanisms back to recollection, and therefore to between-object binding. Comparable to the findings for emotional item memory, there is evidence from ERP studies showing that an emotion-advantage for emotional episodes is reflected in the ERP correlate for recollection of emotional context. For example, as for emotional item-memory, Maratos and Rugg (2001) as well as Ventura-Bort et al. (2016) reported larger parietal old/new effects to emotionally nonvalenced stimuli that were originally encoded together with an emotional background. This can be taken as evidence that item memory for neutral objects can be influenced by emotional contexts by increasing the contribution of recollection, just as it has been proposed for emotional items. For Maratos and Rugg (2001), this may be especially true if encoding of the emotional context was incidental, rather than intentional.

On the other hand, it also is conceivable that emotions modulate item-context binding by increasing the contribution of familiarity-based remembering. In our example above, the bystander’s scream was not central to our encounter with a dog, yet it is still likely that the emotionality of that expression would improve the memory for the event. According to the Object-Based Framework (Mather, 2007), this can occur if emotional arousal is strong enough to shift the attention onto spatially close features by perceptually binding the features of the event together, so that they appear as part of the same object. Arousing components of associations can “grab attention” and enhance within-object binding of contextual information by shifting the attention onto central features (Mather, 2007). In these situations, arousal might therefore improve memory for emotional within-object bindings, because the emotional context will be integrated into the central item. This often is referred to as a process of unitisation (see Murray & Kensinger, 2013, for the role of unitisation in emotion memory). There is behavioural and ERP evidence that unitisation can support memory for item-item or item-context bindings by increasing the contribution of familiarity (Bader et al., 2010; Diana et al., 2008; Jäger et al., 2006). Relating to emotional contexts, Ventura-Bort et al. (2016) reported an increased ERP effect of familiarity (as reflected by the FN400) for objects encoded in emotionally unpleasant, but not for pleasant or neutral contexts. Therefore, one could assume that arousal supports within-object binding, i.e., unitisation, and thus boosts memory performance by increasing familiarity.

Taken together, whilst remembering emotional items appears to be primarily driven by recollection, there is uncertainty to what extend emotional context influences memory for neutral items, and what implications this may have for familiarity and recollection processes. If enhanced memory for items that are integrated with their emotional contexts results from within-object binding and from unitisation during the study phase, this should be evident by modulations of the ERP measure of familiarity, i.e., the mid-frontal old/new effect. On the other hand, if enhanced memory for items in emotional contexts emerges from between-object binding, this should be reflected in modulations of the ERP correlate of recollection (i.e. parietal old/new effect), especially after a longer test-delay. Hence, the present study examines the relative contribution of familiarity and recollection to emotional context memory, immediately and after a 24 h delay.

### Present study

In the present study, we investigated immediate and delayed emotion effects with time-sensitive ERP measures to explore which implications an arousing context during the study phase may have for subsequent familiarity-based and recollection-based item-memory. Different from other studies investigating emotional episodic memories, in this study we compared recognition memory between two separate time-points. This allowed us to investigate how the effects of emotion on recollection unfolded over time by looking at the impact of a 24-h consolidation window on the rate of forgetting of emotional and neutral associations (in line with what has been proposed for item memory by the emotional binding account, Yonelinas & Ritchey, 2015).

The majority of previous studies investigating emotional context memory have largely utilised highly arousing emotional scenes (Lewis et al., 2011; Luck et al., 2014; Ritchey et al., 2013; Smith et al., 2006) or emotional sentences (Maratos & Rugg, 2001) as contextual stimuli. Whilst these stimuli are generally well-suited to elicit emotional responses, the relevance to everyday concepts is somewhat restricted, and, due to ethical considerations, limits the study design to an adult sample. Consequently, it is important to investigate contextual memory based on ecologically valid stimuli that are typically encountered numerously in everyday interactions: neutral and emotionally expressive faces. Another elegant feature of using faces as context stimuli is that the same face-identity can be presented with different emotional face expressions whilst keeping other visual features of the face (i.e., shape or colour) constant. In contrast to other stimulus material, faces elicit specific brain activity: there is evidence that the retrieval of faces compared with names (MacKenzie & Donaldson, 2009) or words (Yick & Wilding, 2008) elicits material-specific brain activity during the same time window as the left-parietal old/new recollection component. Indeed, brain activity during the retrieval of episodic content has been known to differ by the type of source information, such as faces, pictures, or sounds (Peters & Daum, 2009). These findings demonstrate the importance of including face stimuli in recognition memory research to provide a fuller picture of processing source material. Therefore, faces are an ideal stimulus for investigating the contribution of arousal to the memory of item-object associations, and a novel approach in associative recognition memory.

Ideally, one would select faces with multiple emotion expressions as contextual cues, for example one positively and one negatively valenced expression. However, different expressions introduce perceptual variation in facial features. For example, a happy face is usually displayed by expressing an open mouth and showing teeth. Also, because negatively valenced emotions have been found to modulate certain aspects of recognition memory more than positively valenced emotions (Johansson et al., 2004; Ventura-Bort et al., 2016), we selected one unpleasant emotion (i.e., anger) and a neutral face expression, whilst keeping perceptual similarities (i.e., open eyes and closed mouths) consistent across expressions.

In the present study, participants were required to learn neutral objects that were presented in close proximity to either emotional or neutral faces, representing a person thinking about an item. During the study phase, the task was to memorise the face-object associations as one coherent episode to encourage unitisation of features and therefore within-object binding. The encoding of the faces’ emotion-expression was not task-relevant and therefore not intentional. Crucially, each face identity was given an individual three-letter name (i.e., Lea, Max, Tom, Ute). Some studies that have reported emotion effects on context memory also reintroduced emotional cues at test, for example by asking participants to rate rearranged pairs of previously learned emotional associations (Luck et al., 2014). To investigate the effect that emotional context during the study phase may have had on subsequent item memory, it is important to eliminate the reintroduction of emotionality at test to avoid any confounds between the emotion-information stored from the study phase and emotion conveyed by the test-items. As suggested by Maratos and Rugg (2001), an appropriate design for removing this confound is to keep the neutral retrieval cues stable across conditions and to compare neural activity for these cues based on whether they were originally learnt within an emotional or neutral context. Therefore, the present study will utilise the three-letter names given to each face identity as a neutral and ecologically plausible cue for recalling contexts.

Furthermore, the present design utilised a small number of face identities that were repeated numerous times. To ensure that participants did not show arousal-habituation effects, an emotion-induction check for the usefulness of using these face-stimuli was considered necessary to ensure that the stimuli can elicit and maintain an arousal response. An early enhanced frontocentral positivity (P2), as well as the later occurring component Late Positive Potential (LPP) at centroparietal electrodes have previously been found to reflect processing of motivationally relevant stimuli, such as emotional faces (Eimer et al., 2003; Schupp et al., 2000), emotionally unpleasant images (Chen et al., 2018), or emotional scenes, which elicited stable effects across a large number of stimulus repetitions (for a review see Ferrari et al., 2017). If the present emotional face stimuli elicit an arousal response, similarly to traditionally used emotion stimuli, then we would expect to see an emotion effect for the P2 and the LPP during the study phase. Because it has been found that physiological responses to emotional stimuli, such as heart rate, can habituate quickly if repeatedly presented multiple times (Codispoti et al., 2006), this allowed us to explore whether there was an emotion habituation effect when processing these emotion stimuli repeatedly during the study phase.

Assuming that emotional context supports within-object binding, and therefore unitisation, it can be reasoned that this should boost memory performance by increasing the contribution of familiarity-based remembering, as reflected by modulations of the mid-frontal old/new effect. On the other hand, emotional context also may support between-object binding. If this were the case, we would expect enhanced memory for items encountered in an emotional context to be paralleled by the ERP correlate of recollection, i.e., the parietal old/new effect. In this case, we would predict similar levels of familiarity between emotional and neutral context as evident by the mid-frontal old/new effect, the ERP correlate of familiarity. Due to a slower forgetting of emotional content as a result of amygdala-hippocampal interactions during memory formation (Yonelinas & Ritchey, 2015), we expected that the emotion effect for recollection (between-object binding) would increase after a delay of 24 hours.

## Methods

### Participants

Participants were 36 right-handed native German speakers. Vision was normal or corrected-to-normal. For the main analysis, data from four participants were excluded, because they had less than 15 trials in one of the critical conditions after EEG artifact rejection. The final sample for the analysis of the test-phases consisted of 32 participants (aged 18-34 years, *M* age = 23.16, *SD* = 3.95, 21 females), and all participants were screened for medical conditions and drug use. As participants completed two test phases on two consecutive Days 1 and 2 (T1 immediate and T2 after 24 h), we also collected self-reported data on hours of sleep from T1 to T2. Hours slept between both test days ranged from 5 h to 9 h (*M* = 7.03, *SD* = 1.04).

For the study phase, of the initial 36 participants, data from six participants were excluded from analysis if the trial number was below 15, either due to excessive movement during recording (*N* = 3) or due to a technical error with recording triggers in the study phase (*N* = 2).^1^ The final number of participants that contributed to the analysis of the study phase was *N* = 31.

Participants either received €8/h or course credits as compensation. Written informed consent was obtained before the study, which was performed according to the Code of Ethics of the World Medical Association (Declaration of Helsinki) for experiments involving humans. The study was approved by the ethics committee of the Faculty of Human and Business Sciences at Saarland University.

### Stimuli

As contexts served two female (ID 2, 22) and two male (ID 5, 7) face stimuli from the Radboud Face database (Lagner et al., 2010), each depicting either a neutral or an emotional (angry) expression. Stimuli were selected based on validation data with at least 95% emotion recognition accuracy (Lagner et al., 2010). To select stimuli, published valence and intensity ratings for individual expressions were matched across actors (*anger* = intensity: 3.76–3.95, valence: 1.92–2.09; *neutral* = intensity: 3.55–3.88, valence: 2.96–3.50; Lagner et al., 2010). In addition to measuring neurophysiological arousal responses to the faces in the present study, we also collected our own behavioural valence and arousal ratings for each stimulus from a separate follow-up study (*N* = 32) to ensure that the used face stimuli elicited a behavioural as well as neurophysiological response in participants. This follow-up study utilised the same stimuli and study phase, but different test-phases with modified response requirements, and only behavioural data were collected. In addition, participants completed arousal ratings of the faces as part of the study design. The findings are provided as part of the emotion manipulation and habituation analysis in the present results section. For all expressions, mouths were closed and no teeth visible in order to minimise perceptual differences between faces. Faces were cropped to a size of 24 x 39 cm (681 x 851 pixels), and the background colour was light grey. Crucially, each face identity was given an individual three-letter name (Lea, Max, Tom, or Ute). This given name acted as a neutral and ecologically plausible cue for recalling the specific study context of the objects during the test-phase, rather than reintroducing the face-stimuli previously learnt in the study phase.

**Fig. 1:**
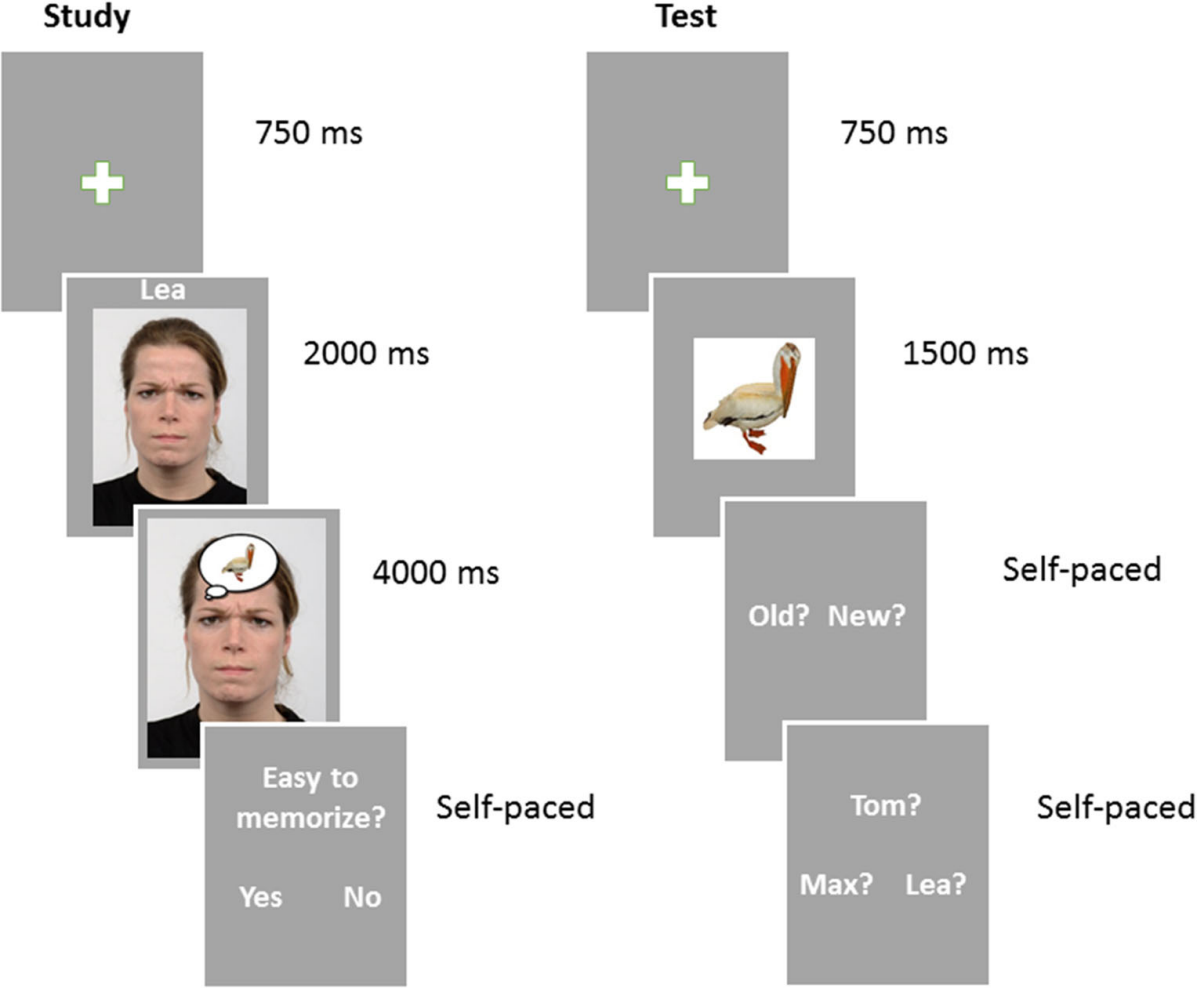
Schematic view of the study and test procedure. *Note.* The contextual name of the actress (Lea) was only shown during the practice session and is added here for illustration purposes. Participants only proceeded to the context judgement task (i.e., name of actor) if they identified the presented object as “old.” The current design consisted of two identical test phases, one immediately and one 24 h after the study phase

Paired with the background faces that served as context, we selected 360 neutral object stimuli taken from the BOSS database (Brodeur et al., 2010; a list of the objects used can be made available upon request). Objects that were inherently American (e.g., yellow school bus) or included letters were deleted in order to adapt the stimuli to a German sample. Objects that had emotional valence (e.g., spider, weapons) or referred to political or religious orientations were excluded. Lastly, images of any human body parts were excluded to reduce potential bias for remembering a human face. The objects were edited to have a white background and were then superimposed within a speech bubble onto the forehead of a background face from one of the four actor identities—to represent a plausible scene in which the actor is thinking about a particular object. This was done to facilitate that the item-context was encoded and memorised as one coherent episode during the study phase via a) a spatially close relationship between object and context, as well as b) a contextually close relationship between the object and context (Figure 1). This face-object pairing was then positioned in the middle of the screen.

In addition, all participants completed a demographics and medical screening form as well as the Edinburgh Handedness Inventory (Oldfield, 1971) before the first test-phase. Furthermore, on both days, participants completed a 19-item self-report scale to assess quality of sleep in both nights prior to testing (Buysse et al., 1989, German version of Pittsburgh Sleep Quality Index), which generally yields strong validity and reliability ratings (Mollayeva et al., 2016).

### Procedure

Participants were seated in front of a 19-inch monitor with a viewing distance of 60-80 cm. All participants completed a brief practice session, including the presentation of six practice face-object pairs. Participants were asked to look at the faces and to learn the names presented alongside the faces to become familiarised with each actor identity and their given three-letter name. This was an important step, because names of actors were not presented during the actual study phase.

#### Study Phase

During the study phase, 240 face-object pairs were shown.^2^ These pairs were counterbalanced for emotionality across participants so that the same object would either appear with an emotional or with a neutral face from the same actor. Each emotional expression (neutral and angry) of each of the three actors was paired with 40 different objects. The resulting 240 object-face combinations were randomly allocated to four study blocks. The presentation of face-object pairs was pseudo-randomised so that no more than three faces of the same emotion or actor identity were shown consecutively. Note that each participant saw images from three out of the four actors only to keep the length and difficulty of the task manageable.

During the study phase, participants were explicitly instructed to memorise the object as well as the face identity by paying attention to the name associated with each of the three face identities. The emotionality of the face expressions was not task-relevant. To ensure that the face-object pairs were actively encoded and memorised as part of one episode, participants’ task in the study phase was to indicate how well they thought they were able to form a mental representation for each pair by pressing 1 for “*easy to memorise*” and 0 for “*not easy to memorise*.”

Each trial started with a white fixation cross, which was presented on a light grey background for 750 ms, followed by a 2-second presentation of either a neutral or an emotional background face. Then, a neutral object was superimposed onto the forehead of each face via a thinking bubble and the face-object pair was shown for an additional 4 seconds.

#### Test Phase

At test, memory was tested for two types of items: objects that were originally encoded in an emotional face context and objects which were originally encoded in a neutral face context. Crucially, recognition memory was tested at two time-points: one immediately after the study phase (T1) and one 24 hours thereafter (T2). Each test-phase included a total of 120 old objects (60 encoded in negative, 60 encoded in neutral context) and 60 new objects. Altogether, we created eight lists of randomised face-object pairings and these were counterbalanced for emotionality, gender of actors, old/new membership, and presentation at test Day 1 (T1) or test Day 2 (T2), using the program Mix (van Casteren & Davis, 2006). For each of the eight lists and within each condition, objects were matched for familiarity, visual complexity and whether ratings for the name given to the object were agreed upon. Independent *t-*tests confirmed that none of these comparisons were significant: all *p* values > 0.05. Participants were then randomly allocated to one of these eight lists.

Old and new objects were randomly allocated to four blocks and the presentation of the objects was pseudo-randomised, with a constraint of maximum repetition of three for old/new objects, as well as the actor presented as background face. On T1, we introduced a delay of 10 minutes between study and the first test-phase in which participants completed a handedness questionnaire and a brief survey on sleep quality concerning the night before the test-Day 1.

Each test phase started with a short practice session in which three old objects from the study phase were presented intermixed with two new objects. The procedure for the practice as well as both test sessions was identical. Each trial started with a fixation cross for 750 ms, followed by either a new or an old object presented for 1,500 ms. In each trial, participants had to indicate whether the seen object was old or new by pressing a corresponding button (right or left arrow), which was counterbalanced across participants. If the object was categorised as “*old*,” a choice of the three actor names from the study phase appeared in the middle of the screen with names randomly distributed across the three possible locations (1 above and 2 below the middle of the screen). The task was to indicate with whom (learnt name of actor) the seen object has previously been paired by pressing a corresponding arrow button pointing to the left, right, or upwards. If the object was categorised as “*new*,” a new trial began.

### ERP Recording and Processing

Electroencephalograms (EEGs) in the study as well as in both test phases were continuously recorded from 64 Ag/AgCl scalp electrodes and attached to the participant’s head based on the extended 10–20 system. The EEG was recorded with BrainVisionRecorder V1.02 (Brain Products, 2012). The EEG was referenced to the left mastoid. Four electrodes placed above and below the left eye as well as on the outer canthi of both eyes measured vertical and horizontal eye movements. Electrical impedance was kept below 5KΩ, and data were filtered with a filter from 0.016 Hz to 100 Hz and sampled at 500 Hz. Data were processed offline with BrainVisionAnalyzer V2.1 (Brain Products, 2012). First, we downsized the sampling rate to 200 Hz and applied a digital high-pass filter with a low cutoff of 0.05 Hz. Then, ocular activity was identified and removed by performing an independent component analysis (ICA). The EEG was re-referenced to the linked mastoids and a low-pass filter was applied with a cutoff at 30 Hz. Finally, for each experimental condition, the EEG was segmented into epochs from −200 ms to 1,500 ms, whereas the first 200 ms before stimulus-onset served as a baseline, which was subtracted from the entire epoch. Event-related potentials (ERPs) were averaged for the study phase and for both test-phases separately.

### Data Analysis

#### Emotion Induction Manipulation Check

##### Behavioural Data

As reported above, participants from a separate follow-up study^3^ (*N* = 32) completed behavioural arousal and valance ratings for each of the eight face stimuli (four emotional, four neutral expressions). Importantly, we collected ratings twice in order to control for potential habituation effects due to multiple repetitions of emotional faces during the study phase. The first rating was completed before the study phase (i.e., before first encounter with the faces) and a second rating was completed after the first test-phase (i.e., after each face was seen 60 times). Faces were presented on a computer screen for 5 seconds each, and participants were instructed to view these faces one-by-one before rating their own perceived emotional arousal as well as their own perceived mood (valence) on a 10-point scale based on the Self-Assessment Manekin (SAM, Lang, 1980). Viewing and rating of the faces was self-paced. Ratings for all emotional and all neutral faces were averaged within each emotion-category and across participants. We conducted two Order (2: first rating, second rating) x Emotionality (2: emotional, neutral) repeated-measures ANOVAs: one for arousal and one for valence ratings.

##### ERP Data

ERPs from the study phase were used to investigate neural responses to processing the face-object pairs during the study phase. In order to check whether the presented emotional object-face stimuli elicited a sufficient neurophysiological emotion-response during the study phase in our participants, we analysed the mean ERPs in the study phase to emotional and neutral faces. The mean trial number and range of each condition that entered analysis of the study phase were as follows: emotional faces: 101.61 (39-119); neutral faces: 101.55 (39-120).

For this, we compared mean amplitudes for the P2 at electrodes C3, Cz, and C4 during an early (170-300 ms), and for the LPP at electrodes CP3, CPz, CP4, P3, Pz, and P4 during a later time-window (400-600 ms) in response to the onset of emotional and neutral face stimuli during the study phase. Both components have previously been found to reflect processing of motivationally relevant stimuli, such as emotional faces (Eimer et al., 2003; Schupp et al., 2000). To control for a further possibility of an emotion-habituation effect during the study phase, we divided the study phase into two halves with the same number of presented emotional (*N* = 60) and neutral stimuli (*N* = 60) in each half. ERPs to mean amplitudes were analysed with separate repeated-measure ANOVAs for each time-window as follows: P2: Emotionality (2: emotional, neutral) x Electrode (3: C3, Cz, C4) x Half (2: first, second); LPP: Emotionality (2: emotional, neutral) x Electrode (6: CP3, CPz, CP4, P3, Pz, P4) x Half (2: first, second).

#### Main Analysis - Emotional item and context memory for T1 and T2

##### Behavioural Data

For each trial during the study phase, participants reported how well they managed to form a mental representation and memorise each face-object pair. A paired samples t-test controlled for an equal percentage of emotional and neutral face-object pairs that were reported to have been memorised easily during the study phase.

Because during the test-phase new items were presented independently of emotionality, only one false-alarm rate could be calculated. Therefore, for item-memory, the proportion of hits as well as reaction times (RT) for hits to objects either originally encoded with a neutral or an emotional background face were analysed (instead of discrimination measures (Pr) which take false alarm rates into account) by two repeated-measures ANOVAs with Emotionality (2: emotional, neutral) x Day (2: T1, T2). For behavioural context-memory, we analysed the proportion of hits for which the correct object (item hits) as well as context (name of the actor) was identified. As such, we calculated (hit + correct context)/hits to quantify context memory. Data were then statistically analysed using a repeated-measures ANOVA with Emotionality (2: emotional, neutral) x Day (2: T1, T2) as factors.

##### ERP Data

To investigate emotion effects of context on item-memory from a dual process perspective, we focused on the mid-frontal old/new effect (300-500 ms) and the left-parietal old/new effect (500-700 ms), the putative ERP correlates of familiarity and recollection, respectively (Diana et al., 2008; Maratos & Rugg, 2001; Smith et al., 2006). Although the typical mid-frontal old/new effect starts around 300 ms post-stimulus, prior studies have suggested that robust old/new effects for emotional images can be found as early as 200 ms post-stimulus (Van Strien et al., 2009). For immediate as well as 24 h delayed testing, Jaeger et al. (2009) have reported more positive-going waveforms for objects paired with emotional scenes from around 200 ms post-stimulus. Based on these reports, we conducted analyses in the 200-400 ms window at F3, Fz, and F4 for familiarity processes and at a later time-window (400-600 ms) at P3, Pz, and P4 for recollection processes. As the number of trials for correct context memory (i.e., correct recall of name associated with the original face-object pair) was not high enough for a reliable analysis (in particular on Day 2), only ERPs for items (old/new judgements) bound to either emotional or neutral contexts were analysed (for a similar approach, see Ventura-Bort et al., 2016). For this, we analysed mean amplitudes for three types of items: emotional hits, neutral hits, and correct rejections of new items, separate for both test days. The mean trial number and range of each condition that entered analysis were as follows: Test Phase 1 (emotional hits: 46.84 [23-58]; neutral hits: 47.63 [28-60]; correct rejections: 53.28 [36-60]), Test Phase 2 (emotional hits: 36.69 [16-49]; neutral hits: 36.63 [18-50]; correct rejections: 49.63 [26-59]).

ERPs were analysed with separate ANOVAs for mean amplitudes in each time-window (200-400 ms at F3, Fz, and F4; 400-600 ms at P3, Pz, and P4) with Type (3: emotional hits, neutral hits, correct rejections of new items) x Day (2: T1, T2) x Electrode (3: F3, Fz, and F4; or P3, Pz and P4) as within-subject factors. Here, we only report results with the hypotheses-relevant factors of* Type *or* Day*.

For all analyses, the significance level was set to *p* = 0.05 and we report the p-values of the two-tailed test. For the within-subject factor *Type*, Mauchly's Test of Sphericity indicated that the assumption of sphericity had neither been violated for the 200-400 ms time-window: χ^2^(2) = 2.09, *p* = 0.35, nor for the 400-600 ms time-window: χ^2^(2) = 1.22, *p* = 0.54. Pairwise comparisons adjusted for multiple comparisons (with Bonferroni-corrected *p*-values) were reported for significant main and interaction effects. As measures of effect-size, we reported partial eta squared (*η*^2^) and Hedge’s g with the average of the two standard deviations as the denominator. It is important to note that throughout, when we report data for emotional or neutral hits, this refers to items originally encoded in emotional or neutral context and therefore items bound to either emotional or neutral background context. The items itself did not carry any emotional valence per se. For all analyses, only correct responses were analysed.

## Results

### Emotion Induction Manipulation Check

In order to report whether there was a sustained arousal elicitation across numerous stimuli repetitions, we first presented the behavioural findings for arousal ratings from the follow-up experiment, followed by mean ERP data for emotional versus neutral faces in the study phase (split into two halves with the same number of stimuli within each category).

#### Behavioural Data

An overview of arousal and valence ratings for emotional and neutral faces at two distinct time-points from the separate follow-up study is given in Table 1. As expected, a repeated-measures ANOVA with emotionality (2: emotional, neutral) and order (2: first rating, second rating) as factors showed that perceived arousal differed significantly for emotionality, *F*(1, 31) = 84.5, *p* < 0.001, *η*^2^ = 0.73, with higher ratings for emotional than neutral faces. There also was a significant order-effect, *F*(1, 31) = 10.49, *p* = 0.003, *η*^2^ = 0.25, with higher overall arousal ratings for the first compared with the second rating. Importantly, emotionality did not interact with this order-effect (*p* = 0.23), demonstrating that although the overall level of arousal decreased from the first to the second rating, the difference between emotional and neutral faces in arousal was not influenced by multiple presentations. Valence ratings also differed significantly for emotional and neutral faces, *F*(1,31) = 237.32, *p* < 0.001, *η*^2^ = 0.88, and emotional (i.e., angry) faces received more negative ratings than neutral faces. There was no significant effect of order (*p* = 0.83) and no interaction between order and emotionality (*p* = 0.81), suggesting that valence ratings of faces were unaffected by habituation.

**Table 1:** Means and standard deviations for arousal and valence ratings for emotional and neutral faces

	Arousal		Valence	
	Emotional faces	Neutral Faces	Emotional faces	Neutral Faces
1^**st**^ **rating** **(before study phase**)	4.14 (0.25)	2.39 (0.22)	1.62 (0.14)	−0.26 (0.13)
2^**nd**^ **rating** **(after first test-phase**)	3.65 (0.27)	2.13 (0.18)	1.62 (0.17)	−0.30 (0.15)

#### ERP Data

**Fig. 2:**
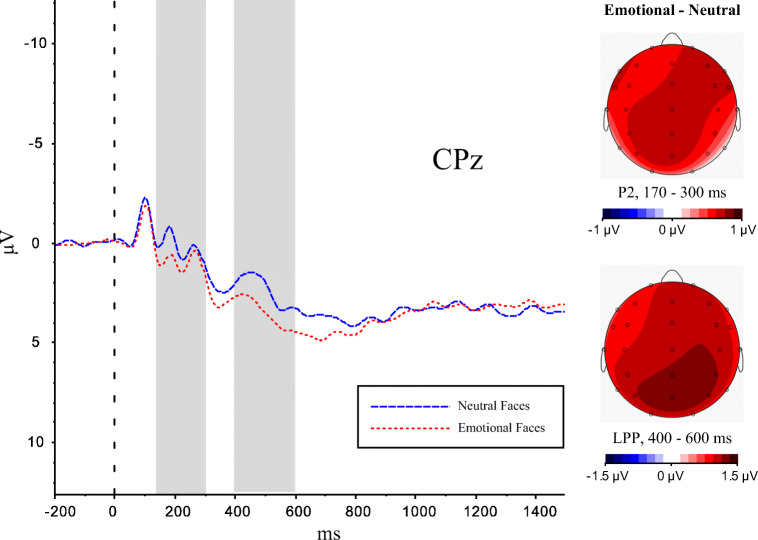
Grand average ERPs (μV) for early P2 (170-300 ms) and late LPP (400-600 ms) emotion effects during the study phase across both halves of the study phase at electrode CPz. Topographic maps showing the pattern of emotional minus neutral differences across the scalp for the same time windows. *Note.* A 12-Hz low pass filter was applied for illustration purposes

The grand average ERP data of the study phase are displayed in Figure 2, for emotional as well as neutral faces. There was a positivity (P2) at central recording sites for emotional faces at around 180 ms after stimulus onset, followed by a second more positive deflection (LPP) starting at around 400 ms at posterior electrodes. The first effect was relatively short (around 50 ms duration) and confined to the central recording sites, whilst the second effect seemed to be longer lasting (approximately 200 ms) and extended over central and parietal recording sites.

##### 170-300 ms

**Fig. 3:**
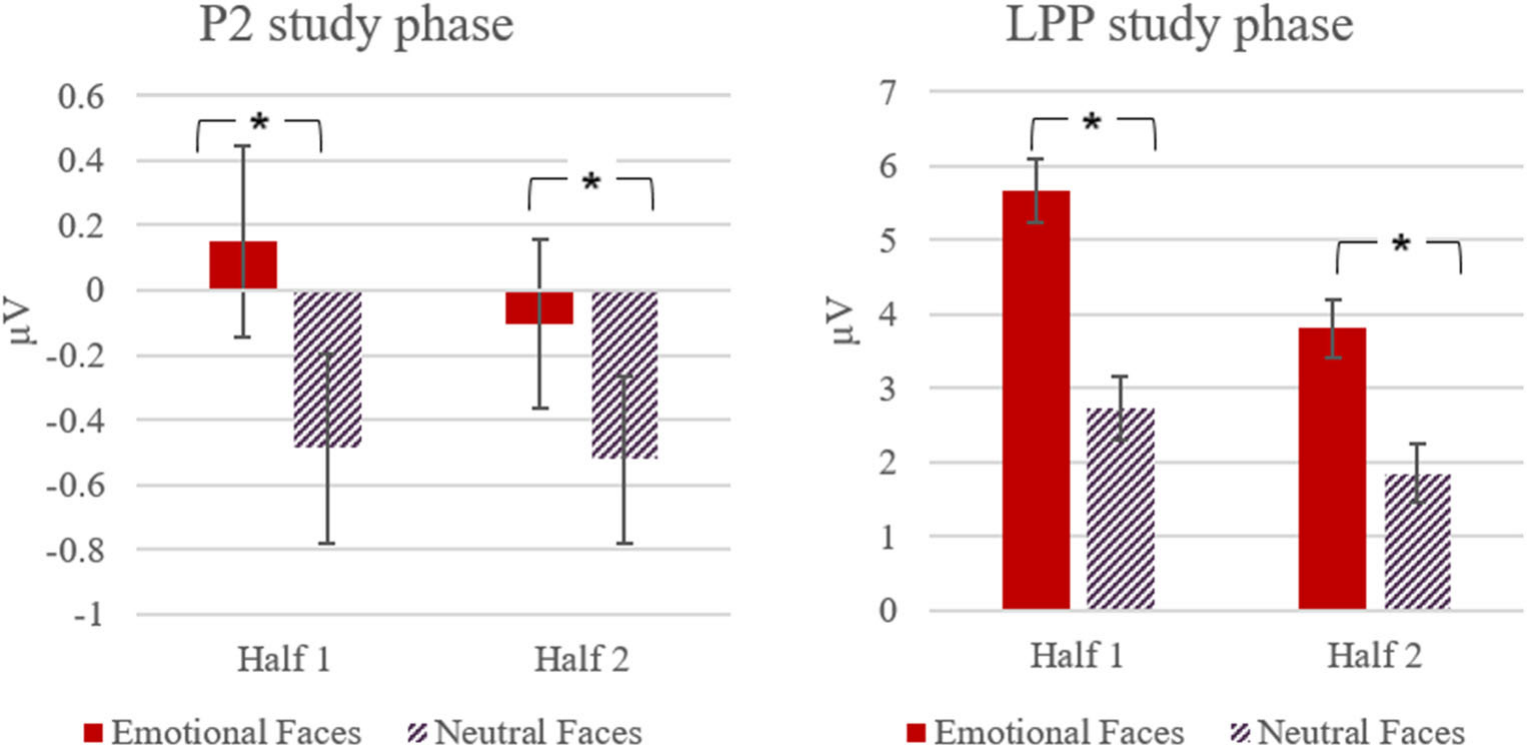
Mean ERPs (μV) and standard errors of the mean difference between emotional and neutral faces at both halves of the study phase for P2 and LPP

For the earlier (P2) time window, a repeated-measures ANOVA with Emotionality (2: emotional, neutral) x Electrode (3: C3, Cz, C4) x Half (2: first, second) revealed a significant main effect of emotionality, *F*(2, 60) = 5.41, *p* = 0.03, *η*^2^ = 0.15, with larger mean amplitudes for emotional (*M* = 0.02, *SE* = 0.64) than for neutral faces (*M* = −0.51, *SE* = 0.61). As shown in Figure 3, we did not obtain a significant main effect for half (*p* = 0.62), and there also was no significant interaction for Emotionality x Half, *p* = 0.49.

##### 400-600 ms

For the later (LPP) time-window, a repeated-measures ANOVA with Emotionality (2: emotional, neutral) x Electrode (6: CP3, CPz, CP4, P3, Pz, P4) x Half (2: first, second) revealed a significant main effect for emotionality, *F*(1, 30) = 16.05, *p* < 0.001, *η*^2^ = 0.35, with more positive mean amplitudes for emotional (*M* = 4.74, *SE* = 0.62) than for neutral faces (*M* = 3.78, *SE* = 0.53). Whilst there was also a significant effect for half, *F*(1, 30) = 15.79, *p* < 0.001, *η*^2^ = 0.35, and stimuli generally elicited more positive going waveforms in the first (*M* = 5.04, *SE* = 0.62) compared with the second half (*M* = 3.47, *SE* = 0.58), this did not interact with emotionality, *F*(1, 30) = 1.44, *p* = 0.24 (Figure 3). These results suggest that whilst ERP responses habituated slightly over time, this applied equally to emotional as well as neutral stimuli and the emotion effect in the LPP remained constant across both halves. Therefore, we concluded that the presented emotional face-stimuli yielded a specific ERP response, as measured in the early P2 and the later LPP during the study phase, and there was no selective habituation effect, suggesting the usefulness of these face-stimuli in the current design.

### Main Analysis – Memory for emotionally-bound items for T1 and T2

#### Behavioural Data

##### Study Phase

For each trial during the study phase, participants’ task was to report how easy they found it to form a mental representation when trying to memorise the face-object pairs. Participants indicated that for 59.5% of emotional pairs, they felt it was easy to form good mental representations and memorise the pairs well compared with 57.8% of neutral pairs. To confirm that the self-reported ability to form mental representations of the face-object associations during the study phase was not driven by the emotionality of the background face, a paired-samples *t*-test confirmed that there was no significant difference between the proportion of easily memorised emotional or neutral face pairs, *t*(29) = 0.83*, p* = 0.41, Hedge’s *g* = 0.1.

##### Test Phase

**Fig. 4:**
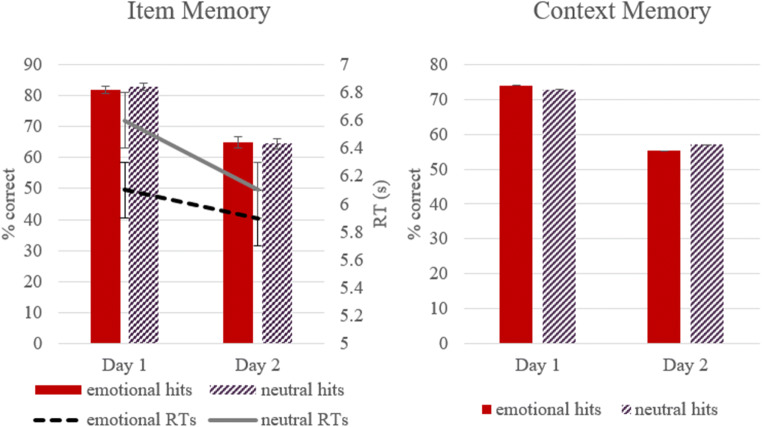
Means and standard errors of the mean difference between emotional and neutral items and context memory for accuracy (%, bars) and reaction times (s, line). *Note.* RTs were only calculated for item memory

For proportion of hits, a repeated-measures ANOVA with Emotionality (2: emotional, neutral) x Day (2: T1, T2) revealed a significant main effect for day, *F*(1, 31) = 114.2, *p* < 0.001, *η*^2^ = 0.79, indicating that more items were correctly remembered on Day 1 than on Day 2. This was independent of context emotionality, as there was no significant main effect for emotionality (*p* = 0.8) and also no significant interactions between day and emotion, *p* = 0.43 (Figure 4). *RTs:* A repeated-measures ANOVA on the reaction times for item hits revealed a significant main effect for emotion, *F*(1,31) = 5.22, *p* = 0.03, *η*^2^ = 0.14, with significantly faster RTs for emotional hits (*M* = 6 s, *SE* = 0.04) compared with neutral hits (*M* = 6.3 s, *SE* = 0.05). There was no significant main effect for day, *p* = 0.25, and emotion did not interact with day of presentation, suggesting faster reaction times for emotional hits on both test-days, *p* = 0.15.

For context memory (i.e., correctly remembered name of face-identity for face-object pair), a repeated-measures ANOVA with Emotionality (2: emotional, neutral) x Day (2: T1, T2) revealed a significant main effect for day, *F*(1, 31) = 84.62, *p* < 0.001, *η*^2^ = 0.73, with a decrease of context memory performance from T1 to T2. There was no significant main effect for emotion, *p* = .82, and also no significant interaction between day and emotion, *p* = 0.4 (Figure 4).

#### ERP Data

In this section, we first present ERP data for the early frontal old/new effect followed by the late parietal old/new effect during the recognition of items that were originally encoded under either emotional or neutral face expression contexts.

##### Early frontal old/new effect (200-400 ms)

**Fig. 5:**
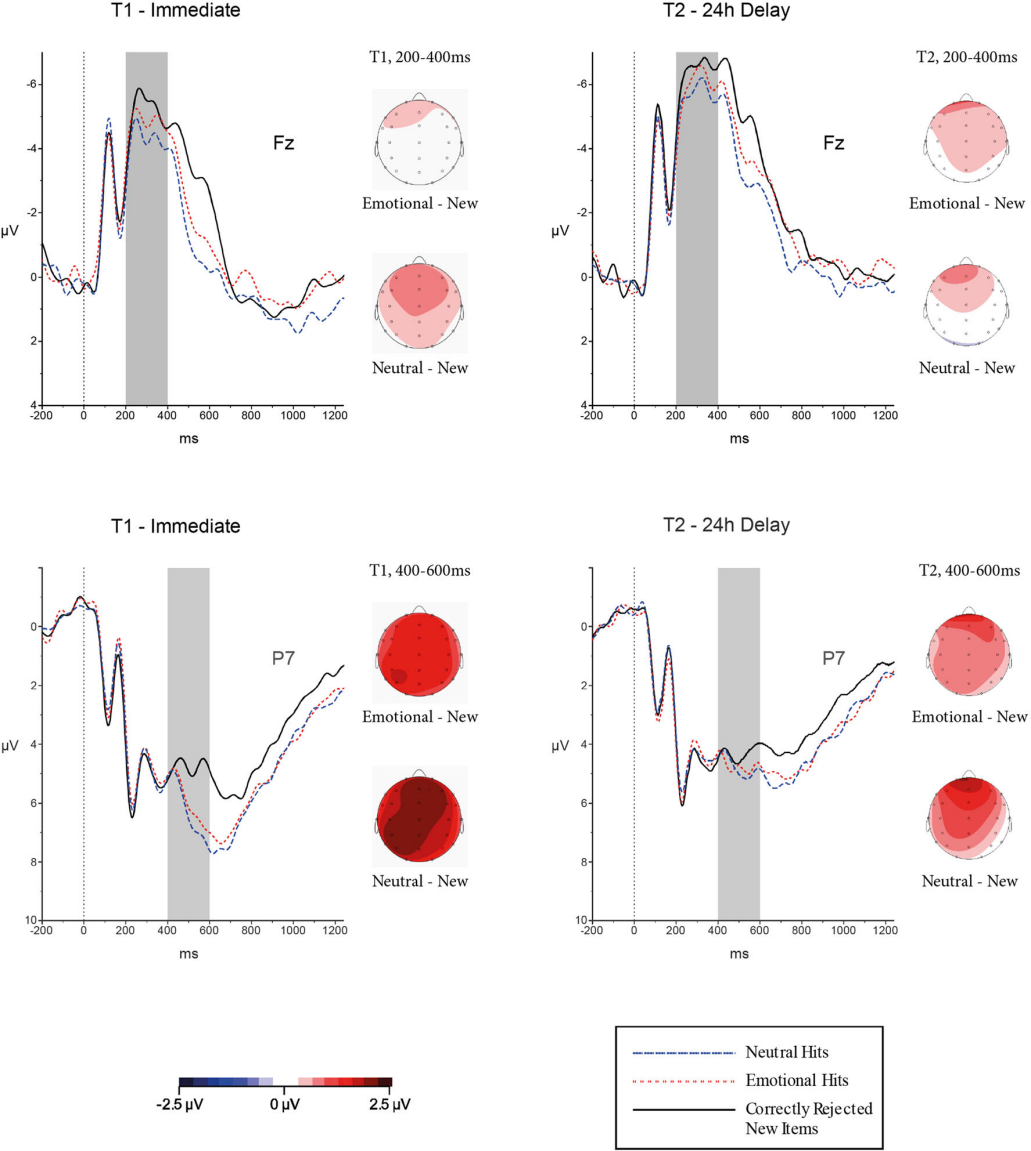
Grand average ERPs (μV) for the early frontal ERP old/new difference (200-400 ms) at electrode Fz and the late central-parietal old/new difference (400-600 ms) at electrode P7. Topographic maps showing the pattern of emotional minus new and neutral minus new differences across the scalp for each time window and delay condition.

Figure 5 (top half) displays the grand average ERPs at electrode Fz for new items, emotional and neutral hits. Overall, the waveforms suggested almost identical activity for items that were encoded under emotional as well as neutral contexts, and this pattern was similar across both days.

For the time-window 200-400 ms at F3, Fz, and F4, results from a repeated-measures ANOVA with Type (3: emotional hits, neutral hits, correct rejections) x Day (2: T1, T2) x Electrode (3: F3, Fz, F4) as within-subject factors, suggested a main effect for type, *F*(2, 62) = 5.87, *p* = 0.005, *η*^2^ = 0.16, and for day, *F*(1, 31) = 16.78, *p* < 0.001, *η*^2^ = 0.35, with more positive mean activity on T1 (*M* = −4.35, *SE* = 0.95) compared with T2 (*M* = −5.58, *SE* = 0.93), *p* < 0.001. Pairwise comparisons adjusted for multiple comparisons (Bonferroni correction) revealed an overall significant old/new effect for items encoded with neutral context, *p* = 0.004, and a marginally significant old/new effect for items encoded with emotional context, *p* = 0.05. There was no significant difference in mean activity to hits originally encoded in emotional or neutral context, *p* = 0.15. There was no significant interaction effect for Type x Day (*p* = 0.84).

##### Parietal old/new effect (400-600 ms)

Figure 5 (lower half) displays the grand average ERPs at electrode P7 for new items, emotional and neutral hits. Overall, the waveforms suggested an old/new effect for all items at Day 1 (T1), irrespective of the emotionality of contextual information. For Day 2 (T2), the old/new effect was generally decreased compared with Day 1.

**Fig. 6:**
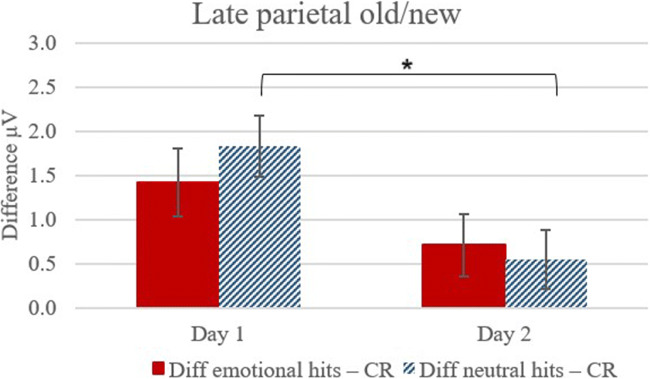
Mean ERPs (μV) and standard error for the old/new differences between emotional hits - correct rejections (CR), and neutral hits - correct rejections (CR) at parietal areas (400-600 ms)

These observations were confirmed by statistical analyses: For the time-window 400-600 ms at P3, Pz, and P4, results from a repeated-measures ANOVA with Type (3: emotional hits, neutral hits, correct rejections) x Day (2: T1, T2) x Electrode (3: P3, Pz, P4) as within-subject factors revealed a main effect for type, *F*(2, 62) = 13.77, *p* < 0.001, *η*^2^ = 0.31, and day, *F*(1, 31) = 29.21, *p* < 0.001, *η*^2^ = 0.49. Importantly, there was also a significant interaction between type and day, *F*(2, 62) = 3.64, *p* = 0.03, *η*^2^ = 0.11. Pairwise comparisons adjusted for multiple comparisons (Bonferroni correction) revealed that for T1 there was an old/new effect for both emotional (*p* = 0.001) and neutral hits (*p* < 0.001). At T2, the old/new effect was close to significant for items originally encoded in emotional context, *p* = 0.058, but not for neutral contexts, *p* = 0.117. Whilst on both days, emotional and neutral hits did not differ from each other significantly (*p* = 0.09, and *p* = 0.67, respectively), two related *t*-tests confirmed that the mean difference between neutral items and correct rejections decreased significantly between Day 1 and Day 2 [*t*(31) = 2.58, *p* = 0.02], which was not evident for the mean difference between emotional items and correct rejections [*t*(31) = 1.39, *p* = 0.17] (see Figure 6 for the mean difference for emotional—correct rejections, and neutral—correct rejections).

## Discussion

The present study looked at delayed contextual emotion effects on item memory to investigate which implications an arousing study context has for subsequent familiarity-based and recollection-based memory. Whereas others have previously looked at the contribution of familiarity and recollection processes to recognition memory for emotional item-context bindings (Mao et al., 2015; Ventura-Bort et al., 2016), to our knowledge this is the first study investigating lasting emotion effects of ecologically valid face expressions on subsequent item memory across two test time-points. More specifically, we tested whether contextual emotion effects conveyed by subtle facial expressions during the study phase could be detected in a recognition memory paradigm across an immediate and 24 h delay test-phase, even if the cue at test was neutral. The behavioural results suggested that, whilst the proportion of correctly recognised objects was not modulated by the context emotionality of faces, participants correctly identified old objects faster when they had been bound to emotional face expressions compared with objects originally encoded in a neutral face context. This was a generic emotion-effect rather than a specific delayed memory-enhancement effect as RT results were not specific to Day 2.

Neurophysiologically, we found an interaction between stimulus type and day for the ERP correlate of recollection with a smaller difference between old and new items on Day 2 compared to Day 1, suggesting that there was less recollection on Day 2. Our data indicated a trend, showing that there was a late central-parietal old/new difference (400-600 ms) for items originally bound to emotional, but not for neutral context faces on Day 2. However, we did not find evidence that the ERP measure of recollection differed significantly between conditions at day 2 (*p* = 0.058).

In addition, no emotion advantage was obtained for the early frontal old/new effect, the ERP correlate of familiarity. Hence, we did not find significant evidence that the early mid frontal effect and the late parietal effect differed as a function of emotional context. Also, for both effects there was no evidence for differential forgetting at Day 2 for items encoded in emotional and in neutral contexts. We suggest that that the present disconnect in effects for RTs and ERPs reflects the possibility that our measures tapped into different underling cognitive processes of memory. The effect of emotion at encoding may cause a nonspecific increase in speed on both days, which corresponds to a facilitated access to long-term storage more generally.

### Neurophysiological underpinnings of emotional item-context bindings

The present findings failed to show significant differences between the emotion conditions. Unlike for item memory, previous research has produced mixed findings on the effect of emotion on associative memory (Bisby & Burgess, 2013; Kensinger, 2009; Mather & Sutherland, 2011; for a review see Dolcos et al., 2017). Especially behavioural findings have reported either enhancing or impairing emotion effects. Those opposing findings have, for example, been discussed by Mather (2007), who proposed that prioritisation of attentional resources leads to enhanced processing of within-object binding (e.g., colourful stimuli) but can impair processing of between-object binding (e.g., two separate superimposed stimuli). However, if the latter has been integrated well enough, this association can be given high attentional priority, and as such emotion facilitates the recall of that association. Our present finding of a similar ERP correlate of familiarity for the neutral and the emotional item-conditions could imply that in both conditions item and contexts were not unitised and therefore the context did not boost familiarity-based remembering. Alternatively, it is possible that item and context were unitised but that the arousal from context faces did not boost unitisation or within-object binding enough in order to enhance the contribution of familiarity for emotional contexts over neutral ones. This is in contrast to our prediction based on the Object-Based Framework (Mather, 2007) which suggested that emotional arousal could support perceptual within-object binding of features by shifting attention to the central parts of the scene.

Contrary to our predictions, we also did not find that emotional context supported between-object binding as we found no significant evidence of enhanced memory for emotional item-context bindings in the parietal old/new effect on Day 2. We can therefore not conclude that contextual emotion conveyed by face expressions encouraged between-object binding and recollection-based remembering of detailed study context.

Recent studies have reported that after a test-delay of 1 week, recollection was higher for neutral items originally encoded with unpleasant in contrast to neutral background scenes, although no immediate recall test was performed, therefore being unable to draw conclusions about how the effects of emotion on recollection unfolded over time (Ventura-Bort et al., 2016, 2019). The present results extend existing research by suggesting that there was no significant emotion-advantage of ecologically valid background faces on subsequent recollection of neutral object stimuli either immediately or after a 24-h delay. Of note, our data indicated a late central-parietal old/new difference (400-600 ms) for items originally bound to emotional (*p* = 0.058), but not neutral context faces on Day 2 (*p* = 0.117). These findings are intriguing because, whilst participants were explicitly instructed to memorise the face-object pairs, at no point during the study was the emotionality of stimuli task-relevant. However, as the difference between emotional and neutral hits failed to reach statistical significance, we will not discuss this any further.

### Disconnect of neurophysiological and behavioural measures

The present data suggest that different aspects of episodic memory were affected by our context manipulation. We reported a nonspecific increase in speed but not memory accuracy, demonstrating facilitated access to emotional memory representations during recognition. Our neurophysiological findings were less conclusive, with no direct evidence for differential forgetting of items encoded in neutral and emotional contexts.

Whilst there is evidence for enhanced memory for emotional items (Kensinger, 2009; Yonelinas & Ritchey, 2015), several studies have previously failed to report a behavioural emotion advantage for stimuli that had been bound to emotional context during the study phase (Fenker et al., 2005; Maratos & Rugg, 2001). This is interesting, because it could be argued that “emotional information might have different functional effects on long-term memory when being inherent to a stimulus as opposed to merely being the context for a neutral stimulus” (Fenker et al., 2005, p. 1998). Often, behavioural emotion effects (i.e., improved performance) have been reported when emotional items were reintroduced at test (Luck et al., 2014) but not if emotional context played a role at encoding only (Fenker et al., 2005; Maratos & Rugg, 2001; Smith et al., 2006).

It is reasonable to assume that, in line with Tulving and Thomson’s (1973) encoding specificity principle, arousal at encoding could make memory traces for items in emotional context at subsequent retrieval more readily accessible (see also Dolcos & Cabeza, 2002). This in turn may be evident in faster reaction times for “old” judgements of these items but may be short of translating to increased accuracy or ERP evidence for preserved recollection at Day 2. We may have seen improved accuracy had we asked participants to make their judgements as quick as possible. Second, participants may have been more confident in their “old” judgements if items had originally been bound to emotional context, and this may result in faster reaction times for the subsequent retrieval of these items. As argued by Bennion et al. (2017), the behavioural measure of reaction times might have simply been more sensitive than accuracy measures in picking up residual emotion effects at test—especially after a test-delay of 24 h and for more subtle, task-irrelevant emotions during a demanding encoding task.

As suggested by Murray et al. (2015), the (left) parietal old/new effect is responsive to the level of memory precision. As such, a greater amplitude reflects a greater amount of detailed information recollected (Vilberg et al. 2006, Vilberg & Rugg, 2007). The present disconnect might be explained by the possibility that a more accessible retrieval process for emotional hits, as reflected in faster RTs, does not necessarily imply a more detailed recollection of episodic memory or indeed a larger parietal old/new effect in the emotion condition.

In our present design, participants were asked to recall neutral objects as well as face identities via their associated names. Therefore, a lack of a significant emotion effect on behavioural performance accuracy as well as neurophysiological data could suggest that faster access to memory of emotional faces did not translate to better memory for face identities. However, this does not exclude the possibility that they may have recalled the emotionality of the background face correctly, had we instructed participants to recall the emotion of the background face at test rather than the three-letter name cue. Alternatively, emotion at encoding may enhance the recollection of other study-relevant details (not just emotionality). In future studies, it might be interesting to investigate how the role of design choices translates to findings more generally.

### Advantages of the present design and stimulus choice

What distinguishes our design from other studies is that the emotionality of the present face-expressions was a) not reintroduced at test, b) more subtle than in typically used highly arousing picture scenes, and c) not task-relevant and therefore encoded incidentally. Even though no direct ERP evidence for differential recollection at Day 2 was obtained, we tentatively conclude that even subtle emotional information presented during the study phase can influence recognition of seemingly neutral items up to 24 hours later (as evident in speeded RTs). In the following, we will discuss why we need further research to confirm these preliminary findings.

#### Neutral cues

Due to the design choice of using neutral cues (i.e. the three letter names of face-identities) rather than reintroducing emotion at test, we report that the emotion at encoding had a lasting effect on memory, as evident in the faster RTs, and is therefore likely to reflect the influence of emotional context on subsequent item memory. According to Tulving and Thomson’s (1973) encoding specificity principle, the study contexts need to be accessible during retrieval in order to recall the stored information correctly. As such, any lasting emotion-effects for recollection after 24 h therefore would suggest that the emotional context information from study extended the accessibility of memory-stored object information. However, further research is needed to establish whether variations in the design could lead to significant differences between emotional and neutral items at test, as our data suggested that neither the neurophysiological correlate for familiarity nor recollection were specific to emotionality.

#### Face stimuli

Our behavioural arousal and valence ratings as well as neurophysiological emotion-arousal induction checks confirmed that ecologically valid face-stimuli indeed induced arousal in our participants during the study phase, which offers a promising alternative to highly arousing emotional stimuli in studies exploring emotional context effects. This in turn opens the application of the research paradigm to a wider range of participants, such as children. Importantly, there also was no evidence of emotion habituation effects of either the behavioural arousal ratings or the neurophysiological LPP component from the first to the second half of the study phase—even if the presentation of the same three emotional faces was repeated 60 times. This suggests that participants maintained an emotional arousal response across the numerous repetitions of the same three emotional faces. In support, Codispoti et al. (2006) also reported stable emotion effects in the LPP during the presentation of three highly emotional IAPS picture scenes which were presented up to 60 times, whilst heart rate and skin conductance habituated very quickly. Codispoti et al. (2016) further discussed that the behavioural measure of attention allocation (i.e., reaction times) can decline quickly following repetition of emotional distractors during a task, whilst neural measures, such as the LPP may reflect the mandatory processing of emotion evaluation, which appears to be unaffected by the repetition of emotional distractors. Our data based on the processing of emotional faces during the study phase are consistent with the view expressed by Codispoti et al. (2016).

### Limitations of the present study

As discussed above, there might be several natural explanations for the present lack of specific emotion effects in both accuracy and the neurophysiological correlate for familiarity and recollection. We also note that our experimental design was limited to one delay condition (24 h only). While some authors have previously reported a clear emotion advantage emerging around 24 h after encoding (Sharot & Phelps, 2004; Sharot & Yonelinas, 2008), others did not find emotional memory improvement immediately or 24 h after encoding (Jaeger et al., 2009). The latter is in line with our finding that the parietal old/new effect for recollection at Day 2 did not differ for emotional and neutral hits. This limitation warrants further research to clarify the role that emotion plays on recollection. For example, it is possible that these emotion effects increase in size after an even longer delay, such as 1 week (Ritchey et al., 2008; Ventura-Bort et al., 2016) or even 1 year (Dolcos et al., 2005; Weymar et al., 2011). Indeed, when comparing immediate with 1-week delayed testing, associative memory for negatively valenced emotion pairs was temporarily impaired at immediate recall but enhanced after 1 week compared with positively or neutral associations (Pierce & Kensinger, 2011). Therefore, future studies could compare the role of varying lengths of longer retention intervals on long-term memory consolidation of emotional context as it is possible that emotion maximises memory accuracy and item-context binding after a longer delay.

Second, to investigate emotion, the present study used angry face expressions. We acknowledge that the current findings for angry faces might not be generalisable to different emotion expressions and the chosen stimuli might in fact not generate enough arousal for lasting emotion effects. However, we also note that incorporating a range of emotion expressions into the research design ultimately introduces perceptual variation across stimuli, which would need to be very closely controlled for (i.e., open and closed mouth). As a solution, further studies using faces could vary the degree of dramatical expressions of the same emotion to investigate how arousal within a set of stimuli could affect item and context memory.

Lastly, as the current data were based on the analysis of hit rates and event-related potentials, the current findings might not be comparable to studies that have measured item memory differently. For example, others (Sharot & Yonelinas, 2008) have calculated Pr scores taking into account new lure items (and therefore false alarm rates) that were specific to emotional conditions. Additionally, a paradigm suitable for collecting alternative measures of familiarity and recollection, such as ROC studies, based on confidence ratings or a remember/know paradigm would allow further insights in the relationship between emotional study context and recognition memory at various delays.

## Conclusions

Overall, we propose that our findings contribute to current research on the mixed effects of emotional items on neutral context memory (compare findings for Bennion et al., 2017; Mao et al., 2015; Mather et al., 2009; Sharot & Yonelines, 2008), by suggesting that neither the neurophysiological correlate for familiarity nor recollection were specific to the emotionality of contextual face expressions. However, emotion effects were reflected in RTs, which may suggest that emotional face expressions presented during encoding can influence recognition of seemingly neutral items. Importantly, our data were based on a specific research design where the emotionality of the present face-expressions was not reintroduced at test, more subtle than in typically used highly arousing picture scenes, and not task-relevant.

Our findings indicated that increased speed found for emotional pairs on both test days generally demonstrated facilitated access to items that were originally bound to emotional contexts. The present findings are a basis for further research in areas, such as trauma-research or the research on the formation of interpersonal relations, as they inform us about the interdependence between the presence of subtle emotion expressions at the time of learning and recognition of seemingly neutral cues 24 h later.

## Footnotes

1 As the present study was based on a within-subject design, which yields higher statistical power with low number of subjects and trials than between-subject designs (Gibney et al., 2020), this trial cutoff was considered adequate for the present study. For final mean trial numbers in each condition, see pages 17-18.

2 Due to a technical error, 83% of participants saw 239 rather than 240 face-object pairs during encoding. This was taken into account when calculating the hit rates from the test-phases for these participants.

3 Behavioural results for valence and arousal ratings stem from participants which were part of a separate behavioural follow-up study and did not participate in the main study (*N* = 32, aged 19-29 years, *M* age = 22.37, *SD* = 2.97, 22 females). The study phase (procedure as well as presentation of the face stimuli) in the follow-up study was identical to the presented main study, whereas the test-phase included modified response requirements. In addition, participants completed behavioural arousal and valance ratings for face stimuli.
